# Plasmonic enhanced low-threshold random lasing from dye-doped nematic liquid crystals with TiN nanoparticles in capillary tubes

**DOI:** 10.1038/s41598-017-16359-5

**Published:** 2017-11-23

**Authors:** Yuan Wan, Yashuai An, Luogen Deng

**Affiliations:** School of Physics, Beijing Institute of Technology, Beijing, 100081 China

## Abstract

We report a plasmonic enhanced low-threshold random lasing from dye-doped nematic liquid crystals with titanium nitride (TiN) nanoparticles (NPDDNLC) in capillary tubes. The NPDDNLC sample yields a coherent random laser with about 0.3 nm in the full width at half maximum (FWHM). We find the laser threshold is decreased by introducing the TiN NPs into the dye-doped nematic liquid crystal sample. The laser threshold decreases with increasing the number density of TiN nanoparticles from 5.613 × 10^10^/*ml* to 5.314 × 10^11^/*ml*. We suggest that the low-threshold random laser is caused by the cooperative effect of the recurrent multiple scattering and field enhancement in the vicinity of TiN nanoparticles. The localized electric field near the TiN nanoparticles enhances the energy absorption of the dye and strengthens the fluorescence amplification. Moreover, we provide a new parameter (the relative efficiency of the stimulated radiation photons) to quantify the quality of the random laser, and we give expressions for the wavelength, mode, and whole emission spectrum. Finally, we find the emission spectrum depends strongly on the emission angle and we discuss the reasons. These findings provide a simple and efficient way for the realization of low-threshold random lasers with low cost.

## Introduction

Since Lawandy *et al*. reported the laser action in strongly scattering media in 1994^1^, random lasers have attracted broad attention, not only because of their unique properties such as small size, flexible shape and omnidirectional emission^2–4^, but also because of their potential applications in photonics, optical imaging and bio-medicine^5–8^. Unlike the conventional lasers, the optical feedback in random lasers is caused by recurrent multiple scattering instead of two high reflection mirrors^2,9,10^. In general, random lasers can be coherent or incoherent, depending on whether the optical feedback is resonant feedback (field feedback) or nonresonant feedback (intensity feedback). The combination of recurrent multiple scattering and the light amplification results in the random laser emission. In the past decades, random lasers have been investigated in many randomly dispersive materials including semiconductor powders^11^, human tissues^12^, metallic nanoparticles (NPs)^13,14^, cellulose nanofibers^15^, graphene^16^, dye-doped liquid crystals (DDLCs)^17,18^ and dye-doped polymer-dispersed liquid crystals (DDPDLCs)^19^. Recently, plasmonic enhanced random lasers have been widely studied by dispersing metal NPs, such as gold (Au) and silver (Ag) NPs, in random systems^13,14,20–24^. As for the Au NPs, in 2006, Popov *et al*. reported the effect of Au nanoparticles on the lasing characteristics of Rh6G laser dyes in polymer films^20^. In 2011, Zhang *et al*. demonstrated a new kind of random laser scheme consisted of a bottom layer of randomly distributed Au nanoislands and a top layer of dye-doped polymer^23^. In 2013, Meng *et al*. investigated the random lasing properties of dye solutions suspended with gold-silica core-shell NPs^25^. In 2016, Wang *et al*. demonstrated an electrically controllable plasmonic enhanced coherent random lasing from the dye-doped nematic liquid crystal containing Au nanoparticles^21^. As for the Ag NPs, in 2005, Dice *et al*. studied surface plasmon-enhanced random laser emission from Ag NPs suspended in laser dye^26^. In 2016, Zhang *et al*. studied a low-threshold direction random lasing in a polymer fiber with Ag NPs^24^. Ning *et al*. reported the enhanced random lasing from dye-doped polymer films with different-sized Ag NPs^27^. Zhai *et al*. demonstrated an ultrathin plasmonic random laser, which consists of a polymer membrance embedded with Ag NPs^28^. However, as a new plasmonic material, titanium nitride (TiN) has not been reported in the plasmonic enhanced random laser field.

Titanium nitride (TiN), which is a good alternative to gold and silver for plasmonic effects in the visible and near-infrared (near-IR) ranges, has been studied as plasmonic materials^29,30^. Plasma frequency can be written as *ω*_*p*_ = (4*πne* ^2^/*m*)^1/2^ ^31^, where n expresses the bulk electron density, e is the electronic charge, m is the electronic mass. With the property of high carrier concentration (around 10^22^ *cm* ^−3^)^32,33^, the plasma frequency of TiN exists in visible region. Compared to noble metals, TiN has some significant advantages as plasmonic materials. The optical properties of TiN may be tuned simply by changing the processing conditions^29,30^. TiN has low loss due to the relatively small magnitude of the imaginary parts of the permittivity^34^. Moreover, TiN is compatible with standard CMOS fabrication process^35^. Finally, TiN is inexpensive compared to noble metals. All of the above advantages make TiN a promising alternative plasmonic material in visible and near-infrared regions.

In this article, we experimentally demonstrate that the low-threshold random laser with low cost can be realized by introducing the TiN nanoparticles in dye-doped nematic liquid crystals. We suggest that the low-threshold random laser is caused by the cooperative effect of the recurrent multiple scattering and the field enhancement in the vicinity of TiN nanoparticles. Compared with the sample without TiN nanoparticles, the NPDDNLC sample has a lower threshold and a narrower linewidth. Then, we study the roles of the number density of TiN nanoparticles on the random laser. The threshold decreases with increasing the number density of TiN nanoparticles from 5.613 × 10^10^/*ml* to 5.314 × 10^11^/*ml*. Moreover, we provide a new parameter (the relative efficiency of the stimulated radiation photons) to quantify the quality of the random laser, and we give the expressions for the wavelength, mode and whole emission spectrum. Finally, we find that the emission spectrum depends strongly on the emission angle. The intensity of the emission spectrum is mainly confined in an angle range from 0° to 45°.

## Results and Discussion

**Figure 1:**
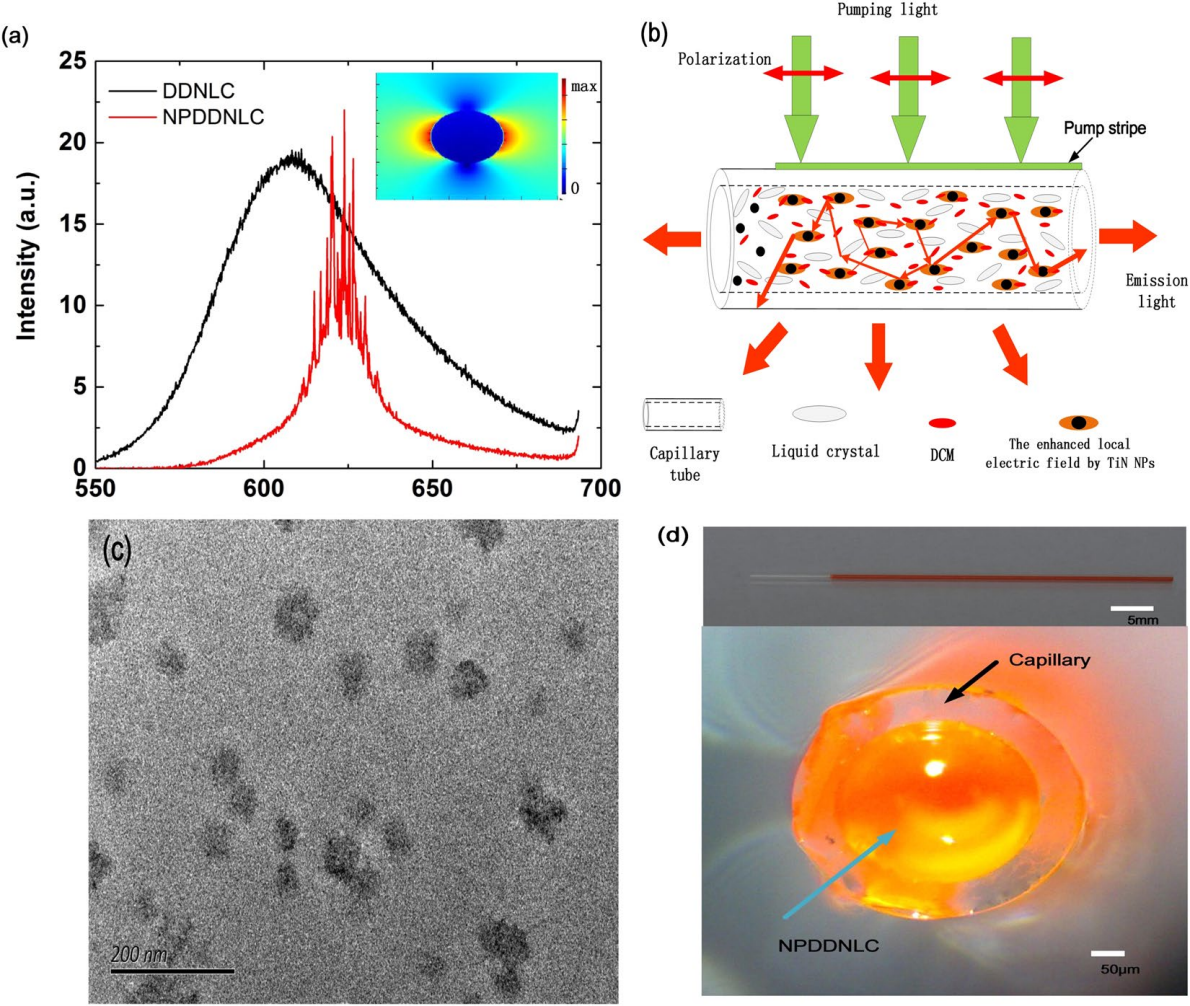
(**a**) Emission spectrum of DDNLC sample (black line) and NPDDNLC sample (red line) with 5.314 × 10^11^/*ml* in TiN NPs number density, when the pump energy is 6.53 μJ/pluse. The inset shows the electric field intensity distribution near the TiN NPs at the wavelength 620 nm simulated by using FDTD methods. (**b**) Schematic illustration of the formation process of the random laser and the description of the recurrent multiple scattering in the capillary tube with NPDDNLC mixtures. (**c**) TEM image of TiN NPs. (**d**) Top: optical photograph of the side view of the capillary tube with NPDDNLC. Bottom: optical micrograph of the front view of the capillary tube with NPDDNLC.

Figure 1(a) shows the emission spectrum of DDNLC sample (black line) and NPDDNLC sample (red line) with 5.314 × 10^11^/*ml* in TiN NPs number density, when the pump energy is 6.53 μJ/pluse. As we can see in Fig. 1(a), when the pump energy is 6.53 μJ/pluse, the emission spectrum of DDNLC sample shows a single broad spontaneous emission peak at the wavelength of about 608 nm. The full width at half maximum (FWHM) of the spontaneous emission spectrum is larger than 60 nm. These mean that the pump energy is lower than the laser threshold energy of the DDNLC sample. While, when the pump energy is 6.53 μJ/pluse, the emission spectrum of NPDDNLC sample shows a lot of discrete sharp peaks above the spontaneous emission at the wavelength from 614 nm to 630 nm. The FWHM of these peaks is less than 0.3 nm, yielding a quality factor Q determined by *λ*/Δ*λ* larger than 2000. These mean that the pump energy is larger than the laser threshold energy of the NPDDNLC sample, and the coherence random laser occurs. Compared with emission spectrum of the DDNLC sample, the emission spectrum of the NPDDNLC sample exhibits a significant red-shift (about 20 nm). This is because the fact that the reabsorption efficiency of the DCM dye is enhanced due to the presence of TiN NPs.

According to the above results, we can draw the conclusion that the laser threshold energy of the DDNLC is decreased by introducing the TiN NPs in the DDNLC sample. We think that TiN NPs enhance the random lasing efficiency through two mechanisms. On the one hand, TiN NPs enhance the electromagnetic field in the vicinity of them due to localized surface plasmon resonance (LSPR). To understand the plasmonic enhancement in such a TiN NP, for the sake of simplicity, we simulated the electric field intensity distribution of a TiN NP with the diameter of 40 nm in vacuum environment at the wavelength of 620 nm by using FDTD methods as shown in the inset of Fig. 1(a). The parameters of TiN are taken from Palik^36^. The mesh size is 1 *nm* × 1 *nm* × 1 *nm*. We can see that the field intensity is enhanced significantly in the vicinity of the TiN NP due to LSPR. On the other hand, the scattering strength is enhanced by adding TiN NPs into the samples, especially at the LSPR wavelength^26^. The formation process of the random laser and the description of the recurrent multiple scattering in the capillary tube with NPDDNLC mixtures are shown in Fig. 1(b). After excitation, the pump light is localized and enhanced around the TiN NPs due to LSPR, which enhances the energy absorption of the dye and strengthens the fluorescence amplification efficiency of the dye near the TiN NPs. The emission light generated from the DCM dye is scattered many times by the NLC molecules and the TiN NPs before escaping from the capillary tube. Because of the recurrent multiple scattering within the capillary tube with NPDDNLC, the diffusion coefficient is efficiently decreased to form spatially localized modes and the dwelling time of the light is increased markedly. When the gain exceeds the loss, the random laser occurs.

**Figure 2:**
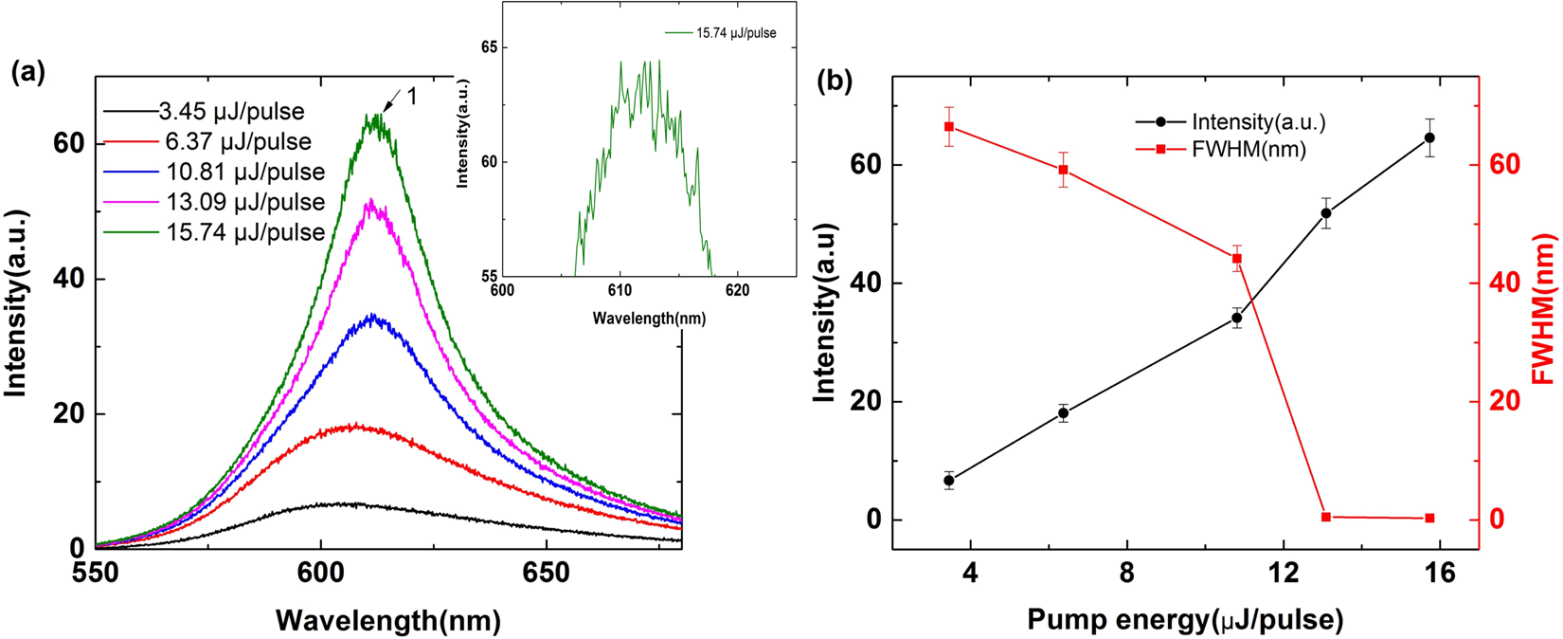
(**a**) The emission spectrum of the DDNLC capillary tube measured at different pump energy. The inset is the enlarged view of the emission spectrum when the pump energy is 15.74 μJ/pulse. (**b**) The peak intensity (black line) and FWHM (red line) of the emission spectrum plotted against pump energy.

For comparison, the emission spectrum of the DDNLC capillary tube at different pump energy is studied as shown in Fig. 2(a). The inset of Fig. 2(a) is the enlarged view of the emission spectrum when the pump energy is 15.74 μJ/pulse. Figure 2(b) shows the peak intensity (black line) and FWHM (red line) of the emission spectrum plotted against pump energy. At low pump energy, the emission spectrum shows a single broad spontaneous emission peak. The intensity of emission spectrum increases with increasing the pump energy. When increasing the pump energy exceeds a given threshold, many discrete sharp peaks can be observed above the spontaneous emission spectrum. As we can see in the inset of Fig. 2(a), the spectral spacing of the adjacent peaks is more or less regular. These peaks are attributed to the random lasing modes^37^. We can obtain that the threshold is about 10.81 μJ/pulse. Here, the threshold is given from both the sharp narrowing of the spectrum and the slope changing of the peak intensity as the pump energy increases. As we can see in Fig. 2(b), when the pump energy exceeds the threshold, the FWHM of the emission spectrum is narrower than 1 nm, which signifies the occurrence of a coherence random laser. The occurrence of random laser is owing to the recurrent multiple scattering caused by the NLC molecules and the internal roughness of the capillary tube.

**Figure 3:**
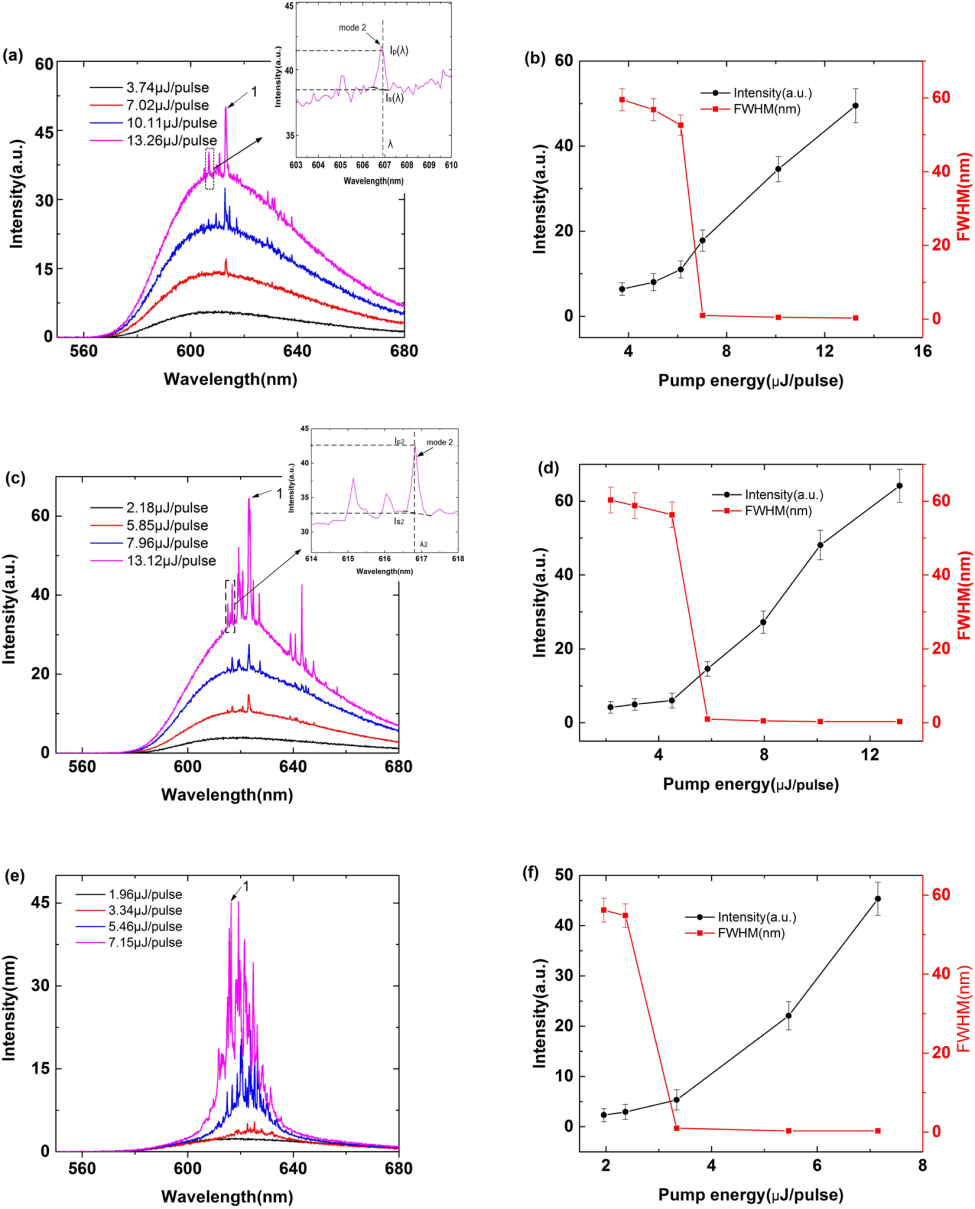
(**a**,**c** and **e**) show the emission spectrum of the NPDDNLC capillary tube measured at different pump energy, when the TiN NPs number density is (**a**) 5.613 × 10^10^/*ml*, (**c**) 1.186 × 10^11^/*ml* and (**e**) 5.314 × 10^11^/*ml*, respectively. (**b**,**d** and **f**) show the peak intensity (black line) and FWHM (red line) of the corresponding emission spectrum plotted against pump energy. The inset in the Fig. 3(a) and the inset in the Fig. 3(c) show the enlarged view of the corresponding square areas in the Fig. 3(a) and Fig. 3(c), respectively.

Figure 3(a,c and e) show the emission spectrum of the NPDDNLC capillary tubes measured at different pump energies, when the TiN NPs number density is 5.613 × 10^10^/*ml*, 1.186 × 10^11^/*ml* and 5.314 × 10^11^/*ml*, respectively. Figure 3(b,d and f) show the peak intensity (black line) and FWHM (red line) of the corresponding emission spectrum Fig. 3(a,c and e) as a function of the pump energy. At low pump energy, the emission spectrum of the NPDDNLC capillary tube shows a single broad spontaneous emission peak (the black line in Fig. 3(a,c and e)). When increasing the pump energy exceeds a given threshold, many discrete sharp peaks are observed above the spontaneous emission spectrum. It is worth pointing out that the laser threshold of the NPDDNLC capillary tubes is much lower than the laser threshold of DDNLC capillary tube. With increasing the number density of TiN NPs from 5.613 × 10^10^/*ml* to 5.314 × 10^11^/*ml*, the laser threshold reduces significantly as shown in Fig. 3(b,d and f). The threshold is about 6.13 μJ/pulse, 4.50 μJ/pulse and 2.37 μJ/pulse when the TiN Nps number density is 5.613 × 10^10^/*ml*, 1.186 × 10^11^/*ml* and 5.314 × 10^11^/*ml*, respectively. The FWHM of the emission spectrum of the NPDDNLC capillary tubes is less than 0.3 nm, which is much narrower than that of the DDNLC capillary tube. The number of the discrete sharp peaks increases with increasing the number density of TiN NPs, when the pump energy well exceeds their threshold. This is because the fact that the fluorescence amplification efficiency of the dye and the recurrent multiple scattering are enhanced by increasing the number density of TiN NPs.

In previous studies^4,21^, the quality factor (*Q* = *λ*/Δ*λ*) is used to characterize the quality of the random cavity. However, only the wavelength and the linewidth of the peak are considered in the quality factor Q, which does not comprehensively characterize the quality of the random laser.

Here, we introduce another new parameter (the relative efficiency *η* of the stimulated radiation photons) to quantify the quality of the random laser. The relative efficiency *η* of the stimulated radiation photons is defined as the ratio between the number of the stimulated radiation photons contributing to random laser and the total number of emission photons. When the frequency of the photons is the same, the light intensity is proportion to the number of photons. Therefore, the relative efficiency *η* of the stimulated radiation photons can be expressed as

1 $$\eta (\lambda )=\frac{{I}_{P}(\lambda )-{I}_{S}(\lambda )}{{I}_{P}(\lambda )},$$

where *I*_*P*_(*λ*) expresses the peak intensity of the emission spectrum at the wavelength *λ*, *I*_*S*_(*λ*) expresses the spontaneous emission intensity at the wavelength *λ* (see Fig. 3(a)). When *η*(*λ*) = 0, there is no random lasing at the wavelength *λ*. When *η*(*λ*) > 0, the random laser occurs at the wavelength *λ*, and the closer to 1 the *η*(*λ*) is, the higher the quality of the random laser is.

For a random laser mode, the relative efficiency *η* of the stimulated radiation photons can be expressed as

2 $${\eta }_{n}={\int }_{{\lambda }_{n1}}^{{\lambda }_{n2}}\frac{{I}_{P}(\lambda )-{I}_{S}(\lambda )}{{I}_{P}(\lambda )}d\lambda ,$$

where n expresses a list of integers marked the random laser modes, *λ*_*n*1_ expresses the starting wavelength of the nth mode, *λ*_*n*2_ is the ending wavelength of the nth mode. Because the random laser mode is very sharp, the equation (2) can be written approximately as

3 $${\eta }_{n}=\frac{{I}_{Pn}({\lambda }_{n})-{I}_{Sn}({\lambda }_{n})}{{I}_{Pn}({\lambda }_{n})+{I}_{Sn}({\lambda }_{n})},$$

where *λ*_*n*_ is the central wavelength of the nth mode, *I*_*pn*_(*λ*_*n*_) is the peak intensity at the wavelength *λ*_*n*_, *I*_*sn*_(*λ*_*n*_) is the spontaneous emission intensity at the wavelength *λ*_*n*_ (see Fig. 3(c)).

For the whole spectrum, the relative efficiency *η* of the stimulated radiation photons can be expressed as

4 $$\eta ={\int }_{{\lambda }_{1}}^{{\lambda }_{2}}\frac{{I}_{P}(\lambda )-{I}_{S}(\lambda )}{{I}_{P}(\lambda )}d\lambda ,$$

where *λ*_1_ is the starting wavelength of the emission spectrum, *λ*_2_ is the ending wavelength of the emission spectrum. The equation (4) can also be written approximately as

5 $$\eta =\frac{\sum _{1}^{n}[{I}_{Pn}({\lambda }_{n})-{I}_{Sn}({\lambda }_{n})]\times FWH{M}_{n}}{{\int }_{{\lambda }_{1}}^{{\lambda }_{2}}{I}_{P}(\lambda )d\lambda },$$

where $\sum _{1}^{n}$ expresses the sum from the mode 1 to mode n, FWHM_n_ expresses the FWHM of the mode n. As we can see in equation (5), for the whole spectrum, the relative efficiency *η* of the stimulated radiation photons is proportion to the number of modes.

We calculate that the relative efficiency *η* of the stimulated radiation photons of the mode 1 at different samples (see Figs 2(a) and 3(a,c and e)) is about 0.03, 0.17, 0.27 and 0.30, respectively. This means that the quality of the random laser is improved by increasing the number density of TiN NPs.

**Figure 4:**
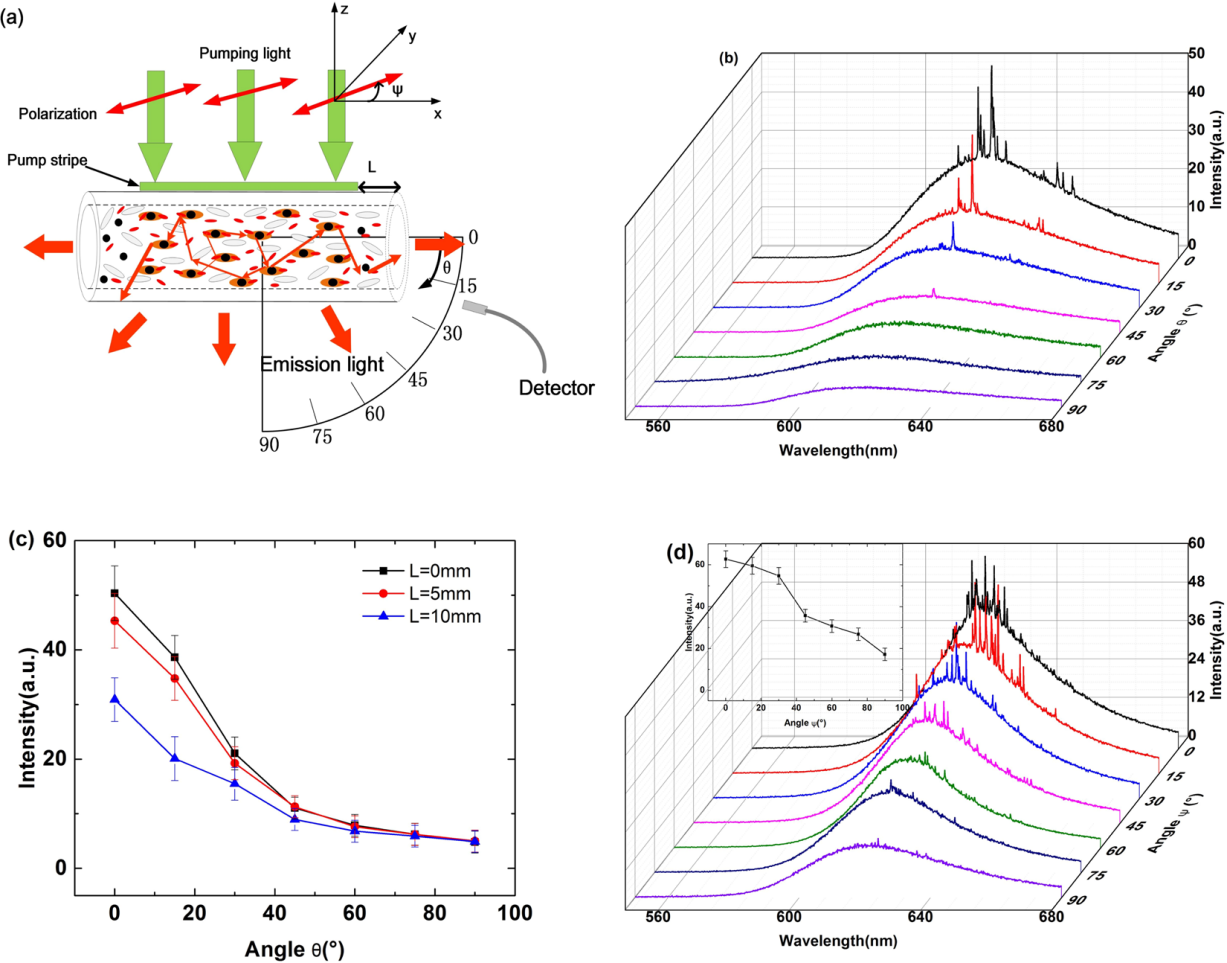
(**a**) The schematic illustration of the emission spectrum detected at different angle. (**b**) The emission spectrum detected at different angle θ from the NPDDNLC sample with 1.186 × 10^11^/*ml* in TiN NPs number density, when L = 0 mm, ψ = 0°. (**c**) The peak intensity of the emission spectrum as a function of the angle θ, when L = 0 mm, 5 mm and 10 mm. (**d**) The emission spectrum of the NPDDNLC sample with 5.314 × 10^11^/*ml* in TiN NPs number density as a function of the polarization direction ψ of the pump beam, when L = 0 mm, θ = 0°. The inset of Fig. 4(d) shows the peak intensity of the emission spectrum as a function of the angle ψ, when L = 0 mm, θ = 0°.

Next, the direction-dependence of the emission spectrum from the NPDDNLC capillary tube is studied. Figure 4(a) shows the schematic illustration of the emission spectrum detected at different angle θ. In Fig. 4(a), the angle θ is defined as the included angle between the detecting direction of the emission spectrum and the direction of the capillary tube. The angle ψ is defined as the included angle between the polarization direction of the pump beam and the direction of the capillary tube. L is the distance between the pump beam and the right side of the capillary tube (see Fig. 4(a)). Figure 4(b) shows the dependence of the emission spectrum on the angle θ, when ψ = 0°, L = 0 mm. As we can see in Fig. 4(b), the emission spectrum strongly depends on the angle θ. When the angle θ is less than 45°, the emission spectrum is random laser with discrete sharp peaks. The discrete sharp peaks decrease sharply with the increase of θ. And, there is only one small peak when θ = 45°. When the angle θ is larger than 45°, the emission spectrum is spontaneous emission. The Fig. 4(c) shows the intensity of the emission spectrum as a function of the angle θ, when L = 0 mm, 5 mm and 10 mm. As we can see in Fig. 4(c), the intensity of the emission spectrum is mainly confined in an angle range from 0° to 45°. The direction-dependence of the emission spectrum decreases with the increase of the distance L. The intensity of the emission spectrum at θ = 45° is 11.04 (a.u.), which is about 22% of the intensity (50.36 (a.u.)) at θ = 0°, when L = 0 mm. However, the intensity of the emission spectrum at θ = 45° is 11.28 (a.u.), which is about 25% of the intensity (45.32 (a.u.)) at θ = 0°, when L = 5 mm. The intensity of the emission spectrum at θ = 45°is 8.96 (a.u.), which is about 28% of the intensity (30.9 (a.u.)) at θ = 0°, when L = 10 mm. This is because the fact that the emission spectrum, detected at the small angle θ, suffers an energy loss before escaping from the capillary tube, when L > 0 mm. In addition, the energy loss increases with the increase of the distance L.

The dependence of the emission spectrum on the angle θ can be explained by four reasons. (1) The capillary tube can be seen as an one-dimensional structure, the light mainly transport along the direction of the capillary tube due to the reflection of the capillary tube. (2) The emission rate of dye molecules, which can be expressed as **E•d**, depends strongly on the coupling of the pump electric field **E** and the transition dipole moment **d** of the dye molecules. Because the direction of the pump electric field **E** is parallel to the capillary tube, the emission rate of dye molecules along the capillary tube is higher than the emission rate of dye molecules along other directions (see Fig. 4(d)). (3) The amplification efficiency of the light transported along the direction of the capillary tube is higher than that perpendicular to the capillary tube due to that the LSPR of the TiN NPs is mainly parallel to the direction of pump electric field **E**. (4) Because the stripe length of the pump light (about 10 mm) is much longer than the stripe width (about 0.2 mm) and the inner diameter of capillary tubes (0.3 mm), the optical gain of the light transported along the long axis of the stripe is much stronger than that along other directions.

## Conclusions

In conclusion, we study the plasmonic enhanced low-threshold random lasing from dye-doped nematic liquid crystals with TiN nanoparticles in capillary tubes. We find that the threshold is lowered and the emission intensity is enhanced by introducing the TiN NPs into DDNLC. The threshold decreases with increasing the number density of TiN nanoparticles from 5.613 × 10^10^/*ml* to 5.314 × 10^11^/*ml*. We suggest that the low-threshold random laser is caused by the cooperative effect of the recurrent multiple scattering and field enhancement in the vicinity of TiN nanoparticles. The localized electric field near the TiN nanoparticles enhances the energy absorption of the dye and strengthens the fluorescence amplification. Moreover, we provide a new parameter (the relative efficiency *η* of the stimulated radiation photons) to quantify the quality of the random laser, and we give the expressions for the wavelength, mode, and whole emission spectrum. Finally, the dependence of the random laser on the emission angle is studied. We find that the coherent random laser is observed in the angle range from 0° to 45°. When the angle exceeds 45°, the random laser quenches. The emission intensity is mainly confined in the angle range from 0° to 45°, and the emission intensity at 45° is only about 22% of the intensity at 0°, when L = 0 mm. These findings provide a simple and efficient way for the realization of low-threshold random lasers with low cost.

## Methods

### Sample Preparation

Because the refractive index and the dielectric tensor of the nematic liquid crystal (NLC) can be controlled by external thermal, electric field and optical field, NLC is extensively used in the investigations of random lasers^4,18,19,21,22^. We employ the NLC (P0616A, refractive indices n_e_ = 1.72 and n_o_ = 1.53 at 20 °C and 589 nm, dielectric constants ε_⊥_ = 5.2 and Δε = 11.5 at 20 °C and 1 kHz) as the host in this experiment. The gain medium used in this experiment is DCM dye provided by Exciton. The TiN NPs with a diameter of about 40 nm are dispersed in the ethanol solution, and the number density ρ of the TiN NPs is 6.289 × 10^11^/*ml*. Firstly, the DCM dye was fully dissolved in the NLC with the ratio of 0.5 wt%: 99.5 wt% to form the dye-doped NLC (DDNLC) mixture. In this step, in order to guarantee that the DCM dye is fully dissolved in the NLC, the mixture was heated to the clearing point and stirred for one hour, and then was cooled to room temperature. Next, we mixed the DDNLC mixture and the ethanol solution of TiN NPs to form four NPs-added DDNLC (NPDDNLC) mixtures with the different number densities of TiN NPs (0, 5.613 × 10^10^/*ml*, 1.186 × 10^11^/*ml* and 5.314 × 10^11^/*ml*). Here, the 0 means that the mixture does not contain TiN NPs. For guaranteeing the dispersity of TiN NPs in the mixtures, an ultrasonic dispersion process was applied in the room temperature. Finally, we immersed the capillary tubes with inner diameter of 300 μm into the four mixtures, respectively. The mixtures were infiltrated into the capillary tubes due to the capillary effect.

### Experimental Set-up

**Figure 5:**
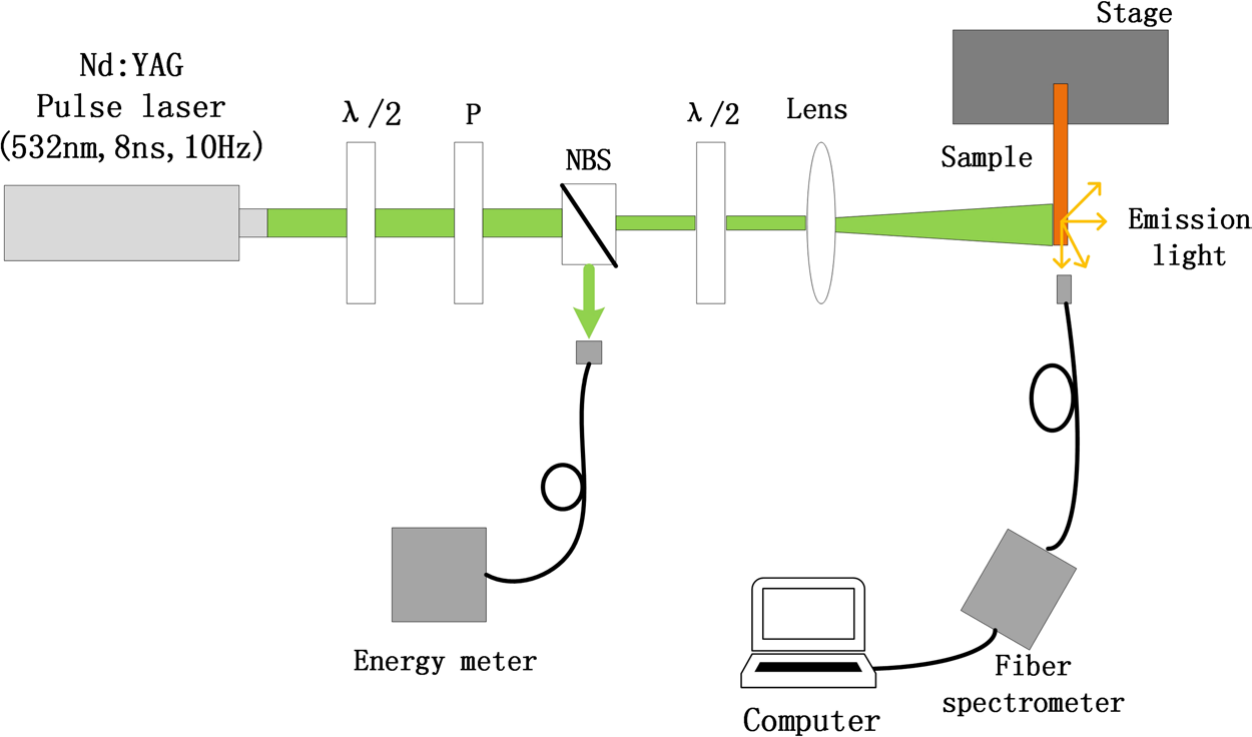
Schematic diagram of the experimental setup.

Figure 5 shows the schematic diagram of the experimental setup. The sample is optically pumped by a frequency doubling Q-switched Nd:YAG laser with 532 nm wavelength, 10 Hz repetition rate, and 8 ns pulse duration. In the Fig. 5(a), *λ*/2, P and NBS is the half-wave plate, polarizer and neutral beam splitter, respectively. When the pump light passes the NBS, it is divided into two sub-beams. One sub-beam, which is reflected by the NBS, is monitored as the reference beam by an energy meter. The other sub-beam as an excitation beam is focused to form an excited stripe (0.2 mm in width and 10 mm in length) on the one side of the capillary tubes by a cylindrical lens (Lens). The polarization of the excitation beam and the long edge of the stripe are all parallel to the capillary tube as shown in Fig. 1(b). The emission spectrum from one side of the capillary tube is collected by a fiber spectrometer with a 0.11 nm in spectral resolution and the emission spectrum is imaged in real time by a computer.
